# Essential Nutrients, Added Sugar Intake, and Epigenetic Age in Midlife Black and White Women: NIMHD Social Epigenomics Program

**DOI:** 10.1001/jamanetworkopen.2024.22749

**Published:** 2024-07-29

**Authors:** Dorothy T. Chiu, Elissa June Hamlat, Joshua Zhang, Elissa S. Epel, Barbara A. Laraia

**Affiliations:** 1 Community Health Sciences Division, School of Public Health, University of California, Berkeley; 2 Osher Center for Integrative Health, University of California, San Francisco; 3 Department of Psychiatry and Behavioral Sciences, University of California, San Francisco; 4 Department of Human Genetics, University of California, Los Angeles

## Abstract

**Importance:**

Nutritive compounds play critical roles in DNA replication, maintenance, and repair, and also serve as antioxidant and anti-inflammatory agents. Sufficient dietary intakes support genomic stability and preserve health.

**Objective:**

To investigate the associations of dietary patterns, including intakes of essential nutrients and added sugar, and diet quality scores of established and new nutrient indices with epigenetic age in a diverse cohort of Black and White women at midlife.

**Design, Setting, and Participants:**

This cross-sectional study included analyses (2021-2023) of past women participants of the 1987-1997 National Heart, Lung, and Blood Institute Growth and Health Study (NGHS), which examined cardiovascular health in a community cohort of Black and White females aged between 9 and 19 years. Of these participants who were recruited between 2015 and 2019 from NGHS’s California site, 342 females had valid completed diet and epigenetic assessments. The data were analyzed from October 2021 to November 2023.

**Exposure:**

Diet quality scores of established nutrient indices (Alternate Mediterranean Diet [aMED], Alternate Healthy Eating Index [AHEI]–2010); scores for a novel, a priori–developed Epigenetic Nutrient Index [ENI]; and mean added sugar intake amounts were derived from 3-day food records.

**Main Outcomes and Measures:**

GrimAge2, a second-generation epigenetic clock marker, was calculated from salivary DNA. Hypotheses were formulated after data collection. Healthier diet indicators were hypothesized to be associated with younger epigenetic age.

**Results:**

A total of 342 women composed the analytic sample (mean [SD] age, 39.2 [1.1] years; 171 [50.0%] Black and 171 [50.0%] White participants). In fully adjusted models, aMED (β, −0.41; 95% CI, −0.69 to −0.13), AHEI-2010 (β, −0.05; 95% CI, −0.08 to −0.01), and ENI (β, −0.17; 95% CI, −0.29 to −0.06) scores, and added sugar intake (β, 0.02; 95% CI, 0.01-0.04) were each significantly associated with GrimAge2 in expected directions. In combined analyses, the aforementioned results with GrimAge2 were preserved with the association estimates for aMED and added sugar intake retaining their statistical significance.

**Conclusions and Relevance:**

In this cross-sectional study, independent associations were observed for both healthy diet and added sugar intake with epigenetic age. To our knowledge, these are among the first findings to demonstrate associations between added sugar intake and epigenetic aging using second-generation epigenetic clocks and one of the first to extend analyses to a diverse population of Black and White women at midlife. Promoting diets aligned with chronic disease prevention recommendations and replete with antioxidant or anti-inflammatory and pro-epigenetic health nutrients while emphasizing low added sugar consumption may support slower cellular aging relative to chronological age, although longitudinal analyses are needed.

## Key Points

**Question:**

Are dietary patterns, including essential nutrients and added sugar intakes, and scores of nutrient indices associated with epigenetic aging?

**Findings:**

In this cross-sectional study of 342 Black and White women at midlife, higher added sugar intake was associated with older epigenetic age, whereas higher essential, pro-epigenetic nutrient intake and higher Alternate Mediterranean Diet (aMED) and Alternate Healthy Eating Index (AHEI)–2010 scores (reflecting dietary alignment with Mediterranean diet and chronic disease prevention guidelines, respectively) were associated with younger epigenetic age.

**Meaning:**

The findings of this study suggest a tandem importance in both optimizing nutrient intake and reducing added sugar intake for epigenetic health.

## Teaser

This cross-sectional study uses data from the NHLBI Growth and Health Study and its follow-up to examine the association of dietary patterns, such as intakes of essential nutrients and added sugar, and scores of nutrient indices with epigenetic age among Black and White women at midlife in the US.

## Introduction

Epigenetic clocks powerfully predict biological age independent of chronological age. These clocks reflect altered gene and protein expression patterns, particularly those resulting from differential DNA methylation (DNAm) at CpG (5′-C-phosphate-G-3′) sites. DNAm that accumulates over time is a testament to the toll social, behavioral, and environmental forces can have on the body.^1,2,3^ These alterations often result in pathogenic processes (eg, genomic instability, systemic inflammation, and oxidative stress) characteristic of aging and chronic disease.^1,4,5^ As such, myriad clocks reflecting epigenetic age have been developed for a range of age- or disease-related targets.^4,6^ The GrimAge series contains second-generation markers of epigenetic aging that account for clinical and functional biomarkers, and is most notable for its robust associations with human mortality and morbidity risk, including time to death and comorbidity counts.^6,7^ The recently developed version 2 of the GrimAge clock (hereafter, GrimAge2) improved on the first’s predictive abilities and confirmed its applicability for people at midlife and of different racial and ethnic backgrounds.^1,6^

Epigenetic changes are modifiable and efforts to counter epigenetic alteration in humans have centered on lifestyle factors including diet, inspiring concepts of an “epigenetic diet” and “nutriepigenetics.”^8,9^ So far, 2 epidemiological studies have found inverse associations between higher diet quality and slower epigenetic aging using clock measures related to mortality, including the first version of GrimAge.^7,10^ In those studies, diet measures were reflective of healthy dietary patterns (eg, the Dietary Approaches to Stop Hypertension [DASH] diet, the Alternate Mediterranean Diet [aMED] score) emphasizing consumption of fruits, vegetables, whole grains, nuts and seeds, and legumes.^8,11^ For example, the Mediterranean-style diet is largely plant-based with emphasis on extra virgin olive oil and seafood. This makes it replete with bioactive nutrients and phytotherapeutic compounds and low in highly processed, high fat, and nutrient-poor foods, a mixture hypothesized to be protective against low-grade chronic inflammation (“inflammaging”), oxidative stress, intracellular and extracellular waste accumulation, and disrupted intracellular signaling and protein-protein interactions. Thus, such a pattern is likely effective in preventing and reversing the epigenetic changes and pathogenic processes associated with aging, disease, and decline.^4,8,12,13,14^

Dietary Reference Intakes (DRIs) are an established set of nutrient-specific reference values determined by experts that guide population intakes for adequacy and toxic effects.^15^ Recent thinking, however, suggests that diets may not always adequately supply nutrients and other bioactives, particularly relative to the amounts necessary to fully condition gene expression or counteract epigenetic alterations to ensure optimal physiological metabolism.^8^ Macronutrients and micronutrients play crucial roles in DNA replication, damage prevention, and repair, whereas nutrient deficiencies (and excesses) can cause genomic damage to the same degree as physical or chemical exposures.^16^ Given that (1) progenome effects of some micronutrients have been observed at different and higher levels than the established DRIs and (2) determination of DRIs does not solely consider genomic stability (ie, lesser susceptibility to genomic alterations), experts have called for refining the DRIs to be better aligned for genomic health maintenance.^14,16,17,18^ Diet quality inventories, such as those for Mediterranean-style diets, have not generally incorporated DRIs, although such considerations could clarify how food-based indices compare against requirements for related nutrients (eg, those with epigenetic properties) and refine epidemiological and intervention efforts. Accordingly, for this study, a novel nutrient index theoretically associated with epigenetic health was created and its associations with epigenetic aging were tested alongside established diet quality indices.

To date, nutriepigenetic work has mostly involved older White populations and focused on healthy dietary aspects. It is therefore important to examine the associations between nutrition and epigenetic aging in more diverse samples and to better understand what specific dietary aspects could be underlying the observed associations. Nutrients with established epigenetic action should be examined, especially considering intakes relative to amounts set forth in the DRIs and nutritional recommendations. Similarly, sugar is an established pro-inflammatory and oxidative agent that has been implicated in cancer as well as cardiometabolic diseases.^19,20,21^ However, in diet quality indices often studied in the epigenetic context (eg, the aMED), sugar is noticeably unaccounted for, and it has also yet to be examined alone. Given the high consumption of sugar globally and the demographic variations within,^22,23,24^ elucidating this association could motivate future dietary interventions and guidelines as well as health disparities research. This study sought to examine associations of diet with GrimAge2 in a midlife cohort comprising Black and White US women. The central hypothesis was that indicators of a healthier diet may be associated with decelerated epigenetic aging, and added sugar intake with accelerated aging.

## Methods

### Data Source and Study Participants

This cross-sectional study used data from the original National Heart, Lung, and Blood Institute (NHLBI) Growth and Health Study (NGHS) (1987-1999) and its follow-up (2015-2019), which studied a cohort of Black and White females aged from 9 or 10 years into midlife (age 36-43 years), examining cardiometabolic health and related determinants. The participants were recruited based on biological female sex at age 9 or 10 years. The follow-up study re-recruited women from the California site.^25,26^ Participants (and/or their parent[s] or guardian[s]) provided demographic data and completed online or paper surveys and new assessments. Participants received remuneration and provided informed consent. The institutional review board of the University of California, Berkeley, approved all study protocols. This study followed the Strengthening the Reporting of Observational Studies in Epidemiology (STROBE) reporting guideline.

For inclusion in current analyses, the participants needed valid diet records and epigenetic data at midlife along with age and race and ethnicity information (participant self-reported); after excluding 5 women with epigenetic data quality issues, 342 individuals were included in the analytic sample. Complete case analyses were done. Among the 624 women who were followed up, the women composing the analytic sample were younger (39.2 years vs 39.9 years; *P* < .001) and had greater body mass index (BMI, calculated as weight in kilograms divided by height in meters squared) compared with women without complete diet and epigenetic data (32.5 vs 30.7; *P* = .02) (Table 1). No differences were otherwise observed.

**Table 1.** Characteristics of 624 Participants Within the Overall Follow-Up Sample, Analytic vs Nonanalytic Sample

Characteristic	Proportions^a^		*P* value of difference
	Analytic sample (n = 342)	Nonanalytic sample (n = 282)	
Current age, mean (SD), y	39.2 (1.1)	39.9 (1.4)	<.001^b^
Race and ethnicity			
Black	171 (50.0)	136 (48.2)	.66
White	171 (50.0)	146 (51.8)	
Annual household income at age 9-10 y, $			
<10 000	60 (17.5)	52 (18.4)	.90
10 000-19 999	58 (17.0)	51 (18.1)	
20 000-39 999	97 (28.4)	76 (27.0)	
≥40 000	110 (32.2)	93 (33.0)	
Missing	17 (5.0)	10 (3.6)	
Highest parental educational attainment at age 9-10 y			
<High school graduate	79 (23.1)	54 (19.2)	.29
Some college	162 (47.4)	129 (45.7)	
College graduate or higher	101 (29.5)	98 (34.8)	
Missing	0	1 (0.4)	
Single parent household at age 9-10 y	119 (34.8)	88 (31.2)	.34
No. of siblings at age 9-10 y, mean (SD)	1.2 (1.1)	1.4 (1.2)	.11
Current BMI, mean (SD)	32.5 (10.0)	30.7 (7.8)	.02^b^
Missing	0	6 (2.1)	.007^b^
Current status of ever smoking			
Yes	150 (43.9)	124 (44.0)	.09
No	192 (56.1)	154 (54.6)	
Missing	0	4 (1.4)	
Chronic health condition diagnosed	164 (48.0)	123 (43.6)	.28
Current medication usage	58 (17.0)	34 (12.1)	.09
Total energy intake, mean (SD), kcal	2019.4 (786.4)	NA	NA
Dietary exposures, mean (SD)			
aMED score	3.9 (1.9)	NA	NA
AHEI-2010 score	55.4 (14.7)	NA	NA
ENI score	13.5 (5.0)	NA	NA
Added sugar, g	61.5 (44.6)	NA	NA
GrimAge2, mean (SD), y^c^	61.3 (5.7)	NA	NA

Abbreviations: AHEI-2010, Alternate Healthy Eating Index 2010 (possible score range, 0-110); aMED, Alternate Mediterranean Diet (possible score range, 0-9); BMI, body mass index (calculated as weight in kilograms divided by height in meters squared); ENI, Epigenetic Nutrient Index (possible score range, 0-24); NA, not applicable.

^a^ Unless otherwise noted, data are given as number (percentage).

^b^ Statistically significant result (*P* < .05).

^c^ Epigenetic clock measure.

### Epigenetic Clock: GrimAge2

Participants provided saliva samples used for DNAm analyses performed by the University of California, Los Angeles Neuroscience Genomics Core (UNGC) of the Semel Institute for Neuroscience and Human Behavior using the Infinium HumanMethylation450 BeadChip platform (Illumina, Inc). DNAm profiles were generated by Horvath’s online calculator,^27^ which provided (1) estimates of epigenetic age based on GrimAge2 estimation methods; and (2) assessments of data quality (again, 5 observations did not pass quality checks). GrimAge2 uses Cox proportional hazards regression models that regress time to death (due to all-cause mortality) on DNAm-based surrogates of plasma proteins, a DNAm-based estimator of smoking pack-years, age, and female sex. It was updated from GrimAge, version 1^6^ by including 2 new DNAm-based estimators of plasma proteins—high-sensitivity C-reactive protein (logCRP) and hemoglobin A_1c_ (log A_1c_)—beyond the original 7. Linear transformation of results from these models allows GrimAge2 to be taken as an epigenetic age estimate (in years). Further information can be accessed from studies on DNA treatment and isolation and advanced analysis options for generating output files^28^ or GrimAge2.^1^

### Dietary and Nutritional Assessment

The participants were instructed by the NGHS study staff to self-complete a 3-day food record at follow-up for 3 nonconsecutive days.^29^ Data were entered into and analyzed by the Nutrition Data System for Research (NDSR) software, version 2018 (University of Minnesota Nutrition Coordinating Center).

### Diet Quality Indices: aMED and AHEI-2010

Mean nutrient and food intakes were calculated across valid food records for each woman based on the NDSR 2018 output. These values were used to calculate the scores of 2 overall diet quality nutrient indices (aMED and the Alternate Healthy Eating Index [AHEI]–2010) and a novel index (Epigenetic Nutrient Index [ENI]) score as described below. The aMED (Mediterranean-style diet) followed published scoring methodology^30^ reflecting the degree of adherence to 9 components of an anti-inflammatory, antioxidant-rich diet. The AHEI-2010 was assessed following published scoring instructions^31^ and reflects the degree of adherence to 11 dietary components associated with decreased risk for chronic disease.

### Epigenetic Nutrient Index

This study developed a novel nutrient index (ENI) after the Mediterranean-style diet, but via a nutrient-based approach rather than a food-based one. Nutrient selection was done a priori based on antioxidant and/or anti-inflammatory capacities as well as roles in DNA maintenance and repair documented in the literature.^16,32,33^ Scores can range from 0 to 24, with higher scores reflecting higher DRI adherence (Table 2).^34^ The internal consistency of the ENI was acceptable (Cronbach α = 0.79). The ENI also demonstrated convergent validity with *r* = 0.51 ENI-aMED correlation as well as higher ENI scores in women from childhood households with higher annual incomes (13.9 vs 11.7, for ≥$40 000/y vs <$10 000/y, respectively) and parental educational attainment (14.7 vs 12.3, for ≥college graduate vs < high school graduate, respectively), corresponding to the literature.^36^ Pearson correlations between the ENI and diet scores and added sugar intake were also calculated. The ENI score was moderately correlated with the AHEI-2010 score (*r* = 0.44) but not correlated with added sugar intake. The aMED and AHEI-2010 scores were highly correlated at *r* = 0.73. Added sugar intake had moderate correlation with the AHEI-2010 score (*r* = −0.44) and low correlation with the aMED score (*r* = −0.28).

**Table 2.** Scoring Rubric for the ENI as Based on the Dietary Reference Intakes^a^

Nutrient	Criteria for 0 points (minimum score)	Criteria for 1 point	Criteria for 2 points (maximum score)
Vitamin A (total activity in retinol equivalents), μg/d^b^	<EAR (500)	EAR ≤ *x* < RDA	≥RDA (700)
Vitamin C (total ascorbic acid), mg/d	<EAR (60)	EAR ≤ *x* < RDA	≥RDA (75)
Vitamin E (total α-tocopherol), mg/d	<EAR (12)	EAR ≤ *x* < RDA	≥RDA (15)
Folate (total folate), μg/d^c^	<EAR (320)	EAR ≤ *x* < RDA	≥RDA (400)
Vitamin B_12_ (total cobalamin), μg/d	<EAR (2)	EAR ≤ *x* < RDA	≥RDA (2.4)
Zinc, mg/d	<EAR (6.8)	EAR ≤ *x* < RDA	≥RDA (8)
Selenium, μg/d	<EAR (45)	EAR ≤ *x* < RDA	≥RDA (55)
Magnesium, mg/d	<EAR (265)	EAR ≤ *x* < RDA	≥RDA (320)
Dietary fiber (total dietary fiber), g/d	<AI/1.2	AI/1.2 ≤ *x* < AI (25)	≥AI (25)
MUFA:SFA (as calories from MUFA:calories from SFA)	<Median_as_	Median_as_ ≤ *x* < 1.2 × median_as_	≥1.2 × Median_as_
Isoflavones (sum of daidzein, genistein, glycitein, biochanin A, and formononetin), mg/d	<Median_as_	Median_as_ ≤ *x* < 1.2 × median_as_	≥1.2 × Median_as_
Total sugar, g/d	≥1.2 × 90 g	90 < *x* < 1.2 × 90	≤90
Sum each component’s score to obtain the total ENI score	0	NA	24

Abbreviations: AI, adequate intake; EAR, estimated average requirement; ENI, Epigenetic Nutrient Index; median_as_, median of the analytic sample; MUFA, monounsaturated fatty acids; NA, not applicable; RDA, recommended dietary allowance; SFA, saturated fatty acids.

^a^ Values corresponding to the EAR, RDA, and AI for each nutrient component are noted (in parentheses) as based on age- and sex-based intake estimates put forth by the Food and Nutrition Board of the US Institute of Medicine and the National Health Service of the United Kingdom (here, values correspond to those for women aged 36-43 years).^34^

^b^ Not accounting for differences in vitamin A activity of carotenoids.^35^

^c^ Not accounting for differences in folate absorption between natural and synthetic forms.^35^

### Added Sugar Intake

Added sugar intake was calculated as the mean across valid food records using NDSR output. The NDSR defines added sugar intake as the total sugar added to foods (eg, as syrups and sugars) during food preparation and commercial food processing. Monosaccharides and disaccharides naturally occurring in foods are not included.^35^

### Covariates

To maximize internal validity and minimize confounding, several covariates were included. Age and sample batch were controlled for as well as naive CD8 and CD8pCD28nCD45Ran memory and effector T-cell counts, thus accounting for normal cell count variation. To control for baseline factors and their potential influence on diet and epigenetic age over time, the following parameters assessed at age 9 or 10 years (mostly parent or caregiver reported) were further adjusted for annual household income, highest parental educational attainment, number of parents in household, and number of siblings. Additionally, self-reported race (Black or White) as well as the current health and lifestyle factors of self-reported chronic conditions (yes to any of the following ever: cancer, diabetes [including gestational, prediabetes], hypertension, or hypercholesterolemia) or medication use (currently yes for any of the following conditions: diabetes, hypertension, hypercholesterolemia, or thyroid), BMI (measured), having ever smoked (yes or no), and mean daily total energy intake (as higher diet quality scores might result from higher energy intake)^37^ were also included.

### Statistical Analysis

Descriptive analyses provided summary statistics. Linear regression models estimated unadjusted and adjusted cross-sectional associations between each of the 4 dietary exposures with GrimAge2. Per expert recommendations, unadjusted models controlled for women’s current age, sample batch, and both naive CD8 and CD8pCD28nCD45Ran memory and effector T-cell counts. Adjusted models controlled for those variables in addition to relevant sociodemographic and health behavior–related covariates already listed. To examine the association between healthy diet measures together with added sugar intake and GrimAge2, aMED, AHEI-2010, and ENI scores were each separately put into the same fully adjusted multivariable linear regression model. The threshold for statistical significance was 2-tailed (α = .05) and all statistical analyses were conducted from October 2021 to November 2023 with Stata15 SE, version 15.1 (StataCorp LLC).

## Results

### Study Participant Characteristics

The analytic sample of this study comprised 342 women (mean [SD] age at follow-up, 39.2 [1.1] years; 171 [50.0%] Black and 171 [50.0%] White participants; mean [SD] BMI, 32.5 [10.0]; 150 [43.9%] ever smokers; 164 [48.0%] ever diagnosed with a chronic condition; and 58 [17.0%] currently taking medication) (Table 1). The participants were well distributed across socioeconomic status categories at baseline (9-10 years old). The participants presented with low to moderate levels of diet quality; the mean (SD) scores were 3.9 (1.9) (possible range, 0-9) on the anti-inflammatory, antioxidant Mediterranean-style pattern (aMED); 55.4 (14.7) (possible range, 0-110) on the AHEI-2010 for chronic disease risk; and 13.5 (5.0) (possible range, 0-24) on the ENI for intakes of epigenetic-relevant nutrients relative to DRIs. The participants also reported mean (SD) daily added sugar intake of 61.5 (44.6) g, although the score range was large (2.7-316.5 g).

### Associations Between Diet and Epigenetic Age

Table 3 provides the overall unadjusted and adjusted associations between each dietary exposure of interest and GrimAge2 resulting from multivariable linear regression models. In both unadjusted and adjusted models, all dietary exposures were statistically and significantly associated with GrimAge2 in the hypothesized, anticipated direction. In adjusted models, the associations observed for each dietary exposure were slightly attenuated. Each unit increase in the scores was associated with year changes in GrimAge2, as follows: aMED (β, −0.41; 95% CI, −0.69 to −0.13), AHEI-2010 (β, −0.05; 95% CI, −0.08 to −0.01), and ENI (β, −0.17; 95% CI, −0.29 to −0.06), indicating that healthier diets were associated with decelerated epigenetic aging. Each gram increase in added sugar intake was associated with a 0.02 (95% CI, 0.01 to 0.04) increase in GrimAge2, reflecting accelerated epigenetic aging.

**Table 3.** Overall Unadjusted and Adjusted Associations Between Dietary Exposure and GrimAge2

Dietary exposure	β (95% CI)	
	Unadjusted (n = 342)^a^	Adjusted (n = 325)^b^
Alternate Mediterranean diet score	−0.62 (−0.91 to −0.34)^c^	−0.41 (−0.69 to −0.13)^c^
Alternate Healthy Eating Index –2010 score	−0.10 (−0.13 to −0.06)^c^	−0.05 (−0.08 to −0.01)^c^
Epigenetic Nutrient Index score	−0.19 (−0.29 to −0.08)^c^	−0.17 (−0.29 to −0.06)^c^
Added sugar, g	0.02 (0.01 to 0.04)^c^	0.02 (0.01 to 0.04)^c^

^a^ Adjusted for factors identified through epigenetic clock best practices and expert recommendations: current age, sample batch, and naive CD8 and CD8pCD28nCD45Ran memory and effector T-cell counts.

^b^ Adjusted for current age, sample batch, naive CD8 and CD8pCD28nCD45Ran memory and effector T-cell counts, race (Black or White), ever diagnosis of a chronic condition, current medication use, baseline (age 9-10 years) household income, baseline (age 9-10 years) highest parental educational attainment, number of parents in household at baseline (age 9-10 years), number of siblings at baseline (age 9-10 years), body mass index at midlife, mean total kilocalories, and ever smoking status assessed at midlife (yes or no). As a complete cases analysis approach was taken, adjusted vs unadjusted analyses differed by n = 17 women with missing baseline household income data.

^c^ Statistically significant result (*P* < .05).

### Associations From Combined Analyses of Diet With Epigenetic Age

Table 4 illustrates the associations of healthy diet measures (aMED, AHEI-2010, and ENI scores) and added sugar intake with epigenetic aging and gives the adjusted results for each healthy diet measure and added sugar intake with GrimAge2 in the context of each other. In all instances, healthier diet measures and added sugar intake appeared to maintain their independent associations with GrimAge2 in the expected directions. Associations were statistically significant for added sugar intake in all models as well as for aMED scores; 95% CIs were more imprecise for AHEI-2010 and ENI scores.

**Table 4.** Overall Adjusted Associations for Diet Quality Scores and Added Sugar With GrimAge2 Where Dietary Exposures Are Combined Among 325 Participants

Dietary exposure model^a^	Adjusted β (95% CI)^b^
Model 1: aMED and added sugar	
aMED score	−0.29 (−0.58 to −0.00)^c^
Added sugar, g	0.02 (0.00 to 0.03)^c^
Model 2: AHEI-2010 and added sugar	
AHEI score	−0.03 (−0.07 to 0.01)
Added sugar, g	0.02 (0.00 to 0.03)^c^
Model 3: ENI and added sugar	
ENI score	−0.12 (−0.25 to 0.01)
Added sugar, g	0.02 (0.00 to 0.03)^c^

Abbreviations: AHEI-2010, Alternate Healthy Eating Index 2010; aMED, Alternate Mediterranean Diet; ENI, Epigenetic Nutrient Index.

^a^ Each dietary exposure model consisted of 1 diet quality score measure (aMED, AHEI-2010, or ENI) (1) plus added sugar (1).

^b^ Adjusted for current age, sample batch, naive CD8 and CD8pCD28nCD45Ran memory and effector T-cell counts, race (Black or White), ever diagnosis of a chronic condition, current medication use, baseline (age 9-10 years) household income, baseline (age 9-10 years) highest parental educational attainment, number of parents in household at baseline (age 9-10 years), number of siblings at baseline (age 9-10 years), body mass index at midlife, mean total kilocalories, and ever smoking status assessed at midlife (yes or no).

^c^ Statistically significant result (*P* < .05).

## Discussion

The findings of this cross-sectional study are among the first, to our knowledge, to demonstrate the association of added sugar intake with an epigenetic clock. Further, to our knowledge, it is the first study to examine the associations of diet with GrimAge2 and extend the applicability of such results to a cohort of Black and White women at midlife. As hypothesized, measures of healthy dietary patterns (aMED, AHEI-2010 scores), and high intakes of nutrients theoretically related to epigenetics (ENI) were associated with younger epigenetic age, while a higher intake of added sugar was associated with older epigenetic age. Additionally, this study examined indicators of healthy and less healthy diets in the same model, allowing simultaneous evaluation of each in the presence of the other. Although the magnitudes of associations were diminished and some 95% CIs became wider, their statistical significance generally persisted, supporting the existence of independent epigenetic associations of both healthy and less healthy diet measures. This approach is informative, as dietary components are often examined singularly or in indices, which can lead to erroneous conclusions if key contextual dietary components are not accounted for or are obscured. From these findings, even in healthy dietary contexts, added sugar still has detrimental associations with epigenetic age. Similarly, despite higher added sugar intake, healthier dietary intakes appear to remain generally associated with younger epigenetic age.

The number of published nutriepigenetic studies, particularly on examining second-generation epigenetic clock markers, is still relatively small. However, the results of the present study are consistent with the literature. Two other studies^7,10^ have examined GrimAge1-associated outcomes and found higher diet quality scores, including the DASH and aMED, were associated with slower epigenetic aging. However, those studies were limited to older (>50 years) and White populations, limiting their demographic generalizability. Analyses of epigenetic aging and added sugar intake are new, but findings are consistent with the larger body of epidemiological work that has drawn connections between added sugar intake and cardiometabolic disease,^19,20^ perhaps suggesting a potential mechanism underlying such observations. Granted, point and 95% CI estimates for the added sugar–GrimAge2 associations were close to zero, suggesting a smaller role for added sugar compared with healthy dietary measures; however, more studies are needed. Nevertheless, their statistical significance was persistent.

Nutrient-based inventories can provide epidemiological contributions for genomic health studies. The idea of epigenetically critical nutrients is important for 2 reasons. First, it supports the notion that epigenetic nutrient intakes above DRI levels could boost epigenetic preservation and potentially motivate updates to nutritional guidelines, an outcome advocated for by nutriepigenetic experts.^16,17,18^ In the novel ENI constructed for the present study, points were awarded based on comparisons of average daily intakes with: (1) estimated average requirements, or the requirement considered adequate for half of the healthy individuals in a population, and (2) recommended dietary allowances or adequate intakes, or where 97% to 98% or essentially all of a population’s healthy individuals’ requirements for a nutrient are met.^15^ Future iterations could test varying ENI scoring parameters relative to DRIs for epigenetic benefit. Second, taking a nutrient approach suggests that any dietary pattern rich in vitamins, minerals, and other bioactives could be useful for preserving epigenetic health. This is helpful because dietary patterns are socioculturally influenced, but a nutrient focus rather than a focus on foods could help bridge cultures, class, and geography.^9^ The Okinawan diet, for example, is nutritionally similar to the Mediterranean-style diet but more aligned to Asian tastes.^38^ In general, the sociodemographic determinants of diet should not be discounted. Across the US population, for instance, it is known that overall diet quality is mediocre and relatively low while added sugar intake is considerably high, as also observed in the sample of the present study. However, specific nutrient intakes will vary based on the particulars of dietary patterns.^22,36^ As dietetics and medicine progresses into the era of personalized nutrition and personalized medicine, the role of social factors including diet will be important to consider in epigenetic studies and could figure prominently in work on health disparities.

### Strengths and Limitations

Strengths of this study are its inclusion of a diverse group of women as well as use of robust measures of diet and DNAm. It was also possible to control for several potential sociodemographic confounders.

This study also has limitations. As a cross-sectional study, it is not possible to infer causality without temporality, and therefore longitudinal studies are needed. Additionally, diet was self-reported via 3-day food records, which may lead to underestimates and overestimates of intakes depending on the nutrient. Therefore, augmenting dietary assessment with food frequency questionnaires and/or biomarkers could be helpful.^39^ Also, other nutrients with pro-epigenetic properties were not included in the current ENI. Still, the Cronbach α for this first ENI version was acceptable at 0.79 and it demonstrated good convergent validity with customary socioeconomic and demographic characteristics. The tolerable upper intake levels of the DRIs were not considered in constructing the ENI. Future work should assess the prevalence of intakes beyond upper limits to assess whether toxicity could be a concern.

## Conclusions

To our knowledge, the findings of this cross-sectional study are among the first to find associations between indicators of healthy diet as well as added sugar intake and second-generation epigenetic aging markers and one of the first to include a cohort of Black women. Higher diet quality and higher consumption of antioxidants or anti-inflammatory nutrients were associated with younger epigenetic age, whereas higher consumption of added sugar was associated with older epigenetic age. Promotion of healthy diets aligned with chronic disease prevention and decreased added sugar consumption may support slower cellular aging relative to chronological age, although longitudinal analyses are needed.

## Notes

Supplement.
Data Sharing Statement
