# Supplementary material for: Essential Nutrients, Added Sugar Intake, and Epigenetic Age in Midlife Black and White Women: NIMHD Social Epigenomics Program
Source: JAMA Netw Open. 2024 Jul 29;7(7):e2422749. doi: 10.1001/jamanetworkopen.2024.22749 (PMC11287388; doi:10.1001/jamanetworkopen.2024.22749)
Supplement: Supplement. — Data Sharing Statement [file jamanetwopen-e2422749-s001.pdf]

Data Sharing Statement

Chiu. Essential Nutrients, Added Sugar Intake, and Epigenetic Age in Midlife Black and White Women. *JAMA Netw Open*. Published July 29, 2024.
doi:10.1001/jamanetworkopen.2024.22749

Data
Data available: No

Additional Information
Explanation for why data not available: Data supporting the findings for this analysis are available by contacting corresponding author DTC and shareable upon reasonable request.
